# Acupuncture Alters Expression of Insulin Signaling Related Molecules and Improves Insulin Resistance in OLETF Rats

**DOI:** 10.1155/2016/9651592

**Published:** 2016-09-22

**Authors:** Xin-Yu Huang, Liang Zhang, Jian Sun, Neng-Gui Xu, Wei Yi

**Affiliations:** ^1^Clinical Medical College of Acupuncture and Rehabilitation, Guangzhou University of Traditional Chinese Medicine, Guangzhou, China; ^2^Guangdong Provincial Hospital of Traditional Chinese Medicine, Guangzhou, China; ^3^Guangzhou University of Traditional Chinese Medicine, Guangzhou, China

## Abstract

To determine effect of acupuncture on insulin resistance in Otsuka Long-Evans Tokushima Fatty (OLETF) rats and to evaluate expression of insulin signaling components. Rats were divided into three groups: Sprague-Dawley (SD) rats, OLETF rats, and acupuncture+OLETF rats. Acupuncture was subcutaneously applied to Neiguan (PC6), Zusanli (ST36), and Sanyinjiao (SP6); in contrast, acupuncture to Shenshu (BL23) was administered perpendicularly. For Neiguan (PC6) and Zusanli (ST36), needles were connected to an electroacupuncture (EA) apparatus. Fasting blood glucose (FPG) was measured by glucose oxidase method. Plasma fasting insulin (FINS) and serum C peptide (C-P) were determined by ELISA. Protein and mRNA expressions of insulin signaling molecules were determined by Western blot and real-time RT-PCR, respectively. OLETF rats exhibit increased levels of FPG, FINS, C-P, and homeostasis model assessment-estimated insulin resistance (HOMA-IR), which were effectively decreased by acupuncture treatment. mRNA expressions of several insulin signaling related molecules IRS1, IRS2, Akt2, aPKC*ζ*, and GLUT4 were decreased in OLETF rats compared to SD controls. Expression of these molecules was restored back to normal levels upon acupuncture administration. PI3K-p85*α* was increased in OLETF rats; this increase was also reversed by acupuncture treatment. Acupuncture improves insulin resistance in OLETF rats, possibly via regulating expression of key insulin signaling related molecules.

## 1. Introduction

Diabetes mellitus (DM) is a significant public health issue worldwide. Methods for controlling plasma glucose levels in patients with DM include diet, exercise, and medication. While most medications are convenient and effective, they may be associated with serious side effects [1, 2]. As such, much research has focused on identifying alternative approaches to increase insulin sensitivity in patients with DM [3–6]. Insulin resistance is the predominant factor contributing to metabolic disorders associated with type 2 diabetes mellitus (noninsulin-dependent, T2DM) [7]. Since T2DM is the most common type of diabetes, improving insulin sensitivity is an important clinical goal.

Traditional Chinese medicine for the treatment of DM includes syndrome differentiation, electroacupuncture (EA), and Chinese massage (Tui na) [3, 8–10]. Much work has shown that EA and Tui na not only decrease plasma glucose levels but also stimulate the neuroendocrine system, the cardiovascular system, and the digestive system to either directly or indirectly regulate plasma glucose [4, 5, 11]. Acupuncture has been practiced in China for thousands of years, and, more recently, it has become a popular therapeutic option for various malignancies in a number of other countries worldwide [12]. Studies have shown that stimulating the CV-12, CV-4, and ST-36 acupoints on both sides of a rat model with a specific frequency significantly reduces plasma glucose levels [4, 13]. Stimulation of acupoints lowers plasma glucose levels to a greater extent than does stimulation of adjacent nonacupuncture points [4]. Numerous studies have demonstrated that acupuncture can correct various metabolic disorders that contribute to the development of insulin resistance, including hyperglycemia, obesity, hyperphagia, hyperlipidemia, inflammation, altered activity of the sympathetic nervous system, and insulin signaling defects [14]. Although acupuncture has the potential to improve pathological conditions [15], the mechanism of the potential effect on insulin resistance remains elusive.

Altered expression or signaling of the insulin signal transduction pathway is a common occurrence associated with insulin resistance. For example, decreased expression of insulin receptor substrate-1 (IRS-1) or reduced serine phosphorylation of this protein is associated with insulin resistance, and, importantly, such alterations influence downstream signaling via phosphatidylinositol-3 kinase (PI3K) [16–18]. These proteins are key molecules involved in insulin signal transduction. Binding of insulin to the insulin receptor (IR) induces autophosphorylation and subsequent tyrosine phosphorylation of docking proteins IRS-1 and IRS-2. Activation of IRS-1 and IRS-2 leads to downstream signal transmission via PI3K. PI3K phosphorylates PIP2 to generate PIP3, which then activates phosphoinositide-dependent kinase (PDK). PDK then activates both Akt (protein kinase B) and protein kinase C (PKC), which influence expression of glucose transporter 4 (GLUT4) [19, 20]. Expression of GLUT4 is important for proper glucose metabolism, particularly in skeletal muscle where it is highly expressed. Within this tissue type, insulin regulates GLUT4 activity to stimulate glucose transport in an Akt/PKC-dependent manner [21, 22].

Otsuka Long-Evans Tokushima Fatty (OLETF) rats exhibit spontaneously elevated blood glucose levels, and, as such, serve as an animal model of type 2 diabetes mellitus (T2DM) [23]. Here, we examine the effect of acupuncture both on insulin resistance in OLETF rats and on the expression of insulin signaling components in rodent skeletal muscle. We find that acupuncture significantly improves insulin resistance in OLETF rats, possibly via regulating expression of key insulin signaling related molecules. These findings suggest that acupuncture may be a valuable treatment option for rats suffering from T2DM.

## 2. Materials and Methods

### 2.1. Animals

Healthy male Sprague-Dawley (SD) rats weighing 222.6 ± 22.6 g and aged 10 weeks were purchased from the Experimental Animal Center of Guangzhou University of Chinese Medicine (China). Male Otsuka Long-Evans Tokushima Fatty (OLETF) rats (body weight: 360.0 ± 35.6 g), genetic models of hyperglycemia that were developed in the Research Laboratory of the Otsuka Pharmaceutical Company [23–25], were supplied from the Research Laboratory of Otsuka Pharmaceutical Company at Hamamatsu (Japan) at 5 weeks of age. Animals were maintained in the Experimental Animal Center of Guangzhou University of Chinese Medicine with free access to water and food for 14 weeks. Animals were housed in Plexiglas cages at a constant room temperature of 20 ± 2°C in a controlled environment and received 12 h of artificial light per day with a relative humidity of 60 ± 10%.

Animals were ethically and humanely treated in accordance with the guiding principles for the Care and Use of Laboratory Animals. The study protocol was approved by the Animal Experimental Committee of Guangzhou University of Chinese Medicine.

### 2.2. Study Design

**Figure 1:**
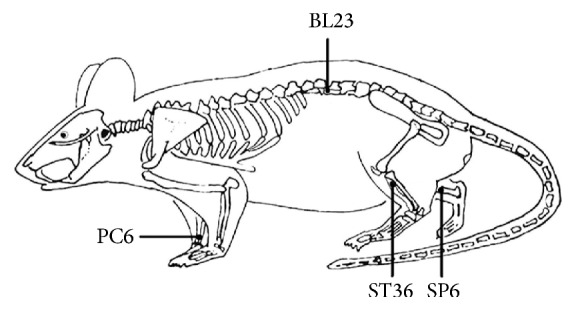
Schematic diagram indicates the four selected acupoints: Neiguan (PC6), Sanyinjiao (SP6), Shenshu (BL23), and Zusanli (ST36); these points correspond to equivalent acupoints in humans.* Neiguan*: located at medialis forelimbs between the radius and ulna, approximately 3 mm from the wrist to the elbow. Regional anatomy: between the deep flexor digitorum, where median nerve and median artery and vein travel through.* Zusanli*: located at the posterolateral knee of hind limbs, about 5 mm below the fibular head. Regional anatomy: between the m. anterior tibialis and m. extensor digitorum longus, where peroneal muscles, peroneal nerve, and anterior tibial artery and vein travel through.* Sanyinjiao*: located at the medial side of the hind leg, 10 mm directly above the tip of the medial malleolus. Regional anatomy: where the flexor digitorum profundus muscle, tibial nerve, and posterior tibial artery and vein travel through.* Shenshu*: located in the lumbar region, at the same level as the inferior border of the spinous process of the second lumbar vertebra (L2). Regional anatomy: where the fascia lumbodorsalis, m. longissimus and m. iliocostalis, hip lumbar artery, lumbar vein, and spinal nerve travel through.

Rats were divided into three groups: control group (SD rats, *n* = 8), hyperglycemic model (OLETF rats, *n* = 8), and acupuncture+OLETF group (*n* = 8). Acupuncture was applied to bilateral Neiguan (PC6), Zusanli (ST36), Sanyinjiao (SP6), and Shenshu (BL23) [26]. Sterilized, disposable, stainless steel acupuncture needles (0.35 × 33 mm) were obtained from Huatuo Medical Devices Co. Ltd. in Suzhou, China. The positions of acupoints described in this study are described in Figure 1.

*Operation*. Neiguan (PC6), Zusanli (ST36), and Sanyinjiao (SP6) were subcutaneously probed at a depth of 8 mm. Shenshu (BL23) was administered perpendicularly at 5 mm. Needles used for Neiguan (PC6) and Zusanli (ST36) were connected to a G6805-2A low-frequency electronic impulse electroacupuncture apparatus (Huayi Medical Instrument Co., Ltd., Shanghai, China); this included administration of sparse-dense waves (sparse wave 2 Hz, dense wave 50 Hz, 2 s, resp.) for 20 min, once per day, for a total of 21 days.

### 2.3. Sampling

All rats were fasted for 12 hours after the last acupuncture treatment. On the second morning, blood samples from the orbital venous sinus were collected. Samples were measured for plasma fasting blood glucose (FPG), plasma fasting insulin (FINS), and serum C peptide (C-P) levels. After blood sampling, rats were anesthetized using pentobarbital sodium (40 mg/kg, intraperitoneal injection). Perfusions were carried out in the presence of 500 IU/mL insulin into the abdominal aorta for all experimental groups; this included a 5 min stabilization period and subsequent perfusion [27]. After perfusions, rats were sacrificed by dislocation, and the musculus quadriceps femoris was rapidly removed and stored at −80°C until real-time RT-PCR and Western blot analysis were performed.

### 2.4. Plasma and Serum Analysis

FPG was measured by the glucose oxidase method. FINS and C-P were determined by enzyme linked immunosorbent assay (ELISA) (kits obtained from R&D Co., USA). The ELISA method was performed according to the manufacturer's instructions. Additionally, the following formula was used to calculate homeostasis model assessment-estimated insulin resistance (HOMA-IR) = FINs (IU/L) × FPG (mmol/L)/22.5. This method has been previously described [28].

### 2.5. Quantitative Real-Time RT-PCR

Total RNA was isolated from musculus quadriceps femoris tissue using TRIzol (Invitrogen, Carlsbad, CA, USA). cDNA synthesis was performed using the MMLV Reverse Transcriptase 1st-Strand cDNA Synthesis Kit (TaKaRa, Dalian, China), according to the directions provided by the manufacturer. Specific mRNA quantification was performed by real-time PCR using SYBR® Green real-time PCR kit (TaKaRa) in a Real-Time PCR Detection System (B&R). The gene-specific primers used for IRS1, IRS2, PI3Kp-85*α*, Akt2, aPKC *ζ*, APKC *λ*, and GLUT4 are shown in Table 1. All reactions involved initial denaturation at 95°C for 2 min followed by 40 cycles of 95°C for 30 s and 60°C for 35 s. The 2^−ΔCt^ method was performed to calculate relative mRNA expression of target genes. *β*-actin was used as an internal control.

### 2.6. Western Blot

Western blot was performed to measure protein expression of PI3Kp85, pPKC *ζ*/*λ*, and GLUT4 in muscle tissue samples. One milliliter of lysis buffer was added per milligram of protein. The tissue sample plus lysis buffer was then homogenized by cryogenic grinding. Pyrolysis was performed at 4°C for 3 hours. The supernatant was collected after centrifugation. For Western blot, equal amounts of protein (30 *μ*g) were separated by 10% SDS-PAGE and electrophoretically transferred to nitrocellulose membrane. Nonspecific binding sites were blocked with 5% milk powder diluted in TBS with 0.05% Tween 20 (TBST) for 60 min. Proteins were detected using the following antibodies: rabbit polyclonal antibody for PI3K-p85 (diluted 1 : 3000; Bios; bs-0128R), rabbit polyclonal antibody for phospho-PKC *ζ*/*λ* (diluted 1 : 3000; CST; #9378), rabbit polyclonal antibody for GLUT4 (diluted 1 : 3000; Boster; BA1626), and mouse monoclonal antibody for GAPDH (diluted 1 : 3000; Boster; BM1623). GAPDH was used as an internal control. Following overnight incubation with the primary antibody at 4°C, each blot was washed three times with TBST buffer. Blots were then incubated with horseradish peroxidase-conjugated secondary antibodies (diluted 1 : 6000; Boster) at room temperature for 60 min. Proteins were detected using an enhanced chemiluminescence reagent. Band intensity was quantified using SensiAnsys gel image analysis software.

### 2.7. Statistical Analysis

Statistical analysis was performed using SPSS analytical software 20.0 (SPSS Inc., Chicago, IL, USA). Data are expressed as mean ± standard deviation (*x* ± *s*). Statistical significance was evaluated by one-way analysis of variance (ANOVA) with Student-Newman-Keuls (SNK) test for post hoc analysis. A *P* value less than 0.05 was considered statistically significant.

## 3. Results

### 3.1. Effects of Acupuncture on Plasma FPG, Plasma FINS, Serum C-P, and HOMA-IR in OLETF Rats

Compared to the SD control group, FPG levels and HOMA-IR were significantly elevated in OLETF rats (both *P* < 0.05). Additionally, FINS and C-P were also increased in the OLETF group compared to SD controls (both *P* < 0.01) (Table 2). When compared to the SD group, levels of FPG and HOMA-IR were significantly decreased in the acupuncture+OLETF group (both *P* < 0.01); in contrast, no significant differences in either FINS or C-P were detected between these two groups (both *P* > 0.05). Levels of FPG, FINS, HOMA-IR, and C-P in the acupuncture+OLETF group were all significantly less than levels in the OLETF group (all *P* < 0.01).

### 3.2. Effects of Acupuncture on mRNA Expression of IRS1, IRS2, PI3Kp-85*α*, Akt2, aPKC*ζ*, aPKC*λ*, and GLUT4 in Skeletal Muscle of OLETF Rats

**Figure 2:**
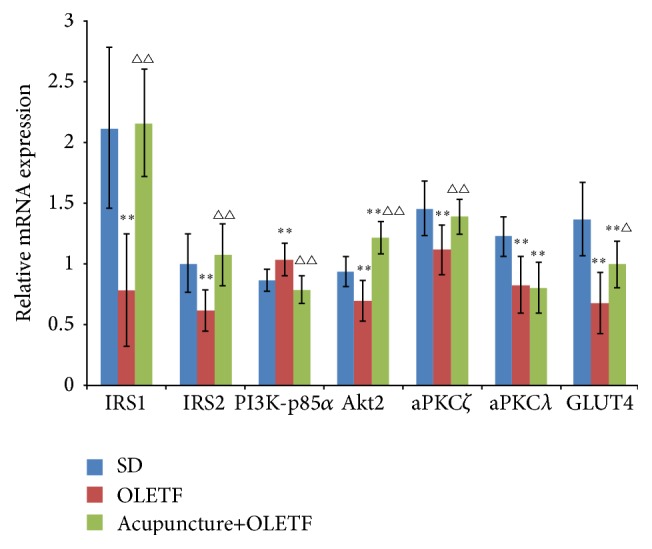
Effects of acupuncture on mRNA expressions of IRS1, IRS2, PI3K-p85*α*, Akt2, aPKC*ζ*, aPKC*λ*, and GLUT4 in skeletal muscle of OLETF rats. Acupuncture was applied to the indicated region for 20 min per day, over the course of 21 days. Following administration, the musculus quadriceps femoris was obtained. Relative mRNA expression was determined by real-time RT-PCR. Data are showed as mean ± standard deviation (SD) (*n* = 8 each group). ^*∗∗*^ *P* < 0.01* versus* SD group; ^△^ *P* < 0.05 and ^△△^ *P* < 0.01* versus* OLETF group. IRS1: insulin receptor substrate 1; IRS2: insulin receptor substrate 2; PI3K: phosphatidylinositol-3 kinase; PKC: protein kinase C; GLUT4: glucose transporter 4; Akt2: protein kinase B beta.

mRNA expression of IRS1, IRS2, Akt2, aPKC*ζ*, aPKC*λ*, and GLUT4 were all significantly reduced in skeletal muscle from OLETF rats compared to skeletal muscle from control SD rats (Figure 2) (all *P* < 0.01). In contrast, mRNA expression of PI3K-p85*α* was significantly higher in OLETF rats compared to controls (*P* < 0.01). mRNA expression of Akt2 was significantly higher in the acupuncture+OLETF group compared to the SD control group (*P* < 0.01). In contrast, mRNA expressions of GLUT4 and aPKC*λ* were reduced in the acupuncture+OLETF group compared to SD controls (both *P* < 0.01); expressions of other genes IRS1, IRS2, PI3K-p85*α*, and aPKC*ζ* were not significantly altered between these two groups (all *P* > 0.05). When comparing the acupuncture+OLETF group to the OLETF group alone, we find that mRNA expressions of IRS-1, IRS-2, Akt2, aPKC*ζ*, and GLUT4 are all significantly elevated in the animals receiving acupuncture treatment (all *P* < 0.05); in contrast, transcript level of PI3K-p85*α* was significantly reduced (*P* < 0.01) upon acupuncture.

### 3.3. Effects of Acupuncture on Protein Expression of PI3Kp85, pPKC *ζ*/*λ*, and GLUT4 in Skeletal Muscle of OLETF Rats

**Figure 3:**
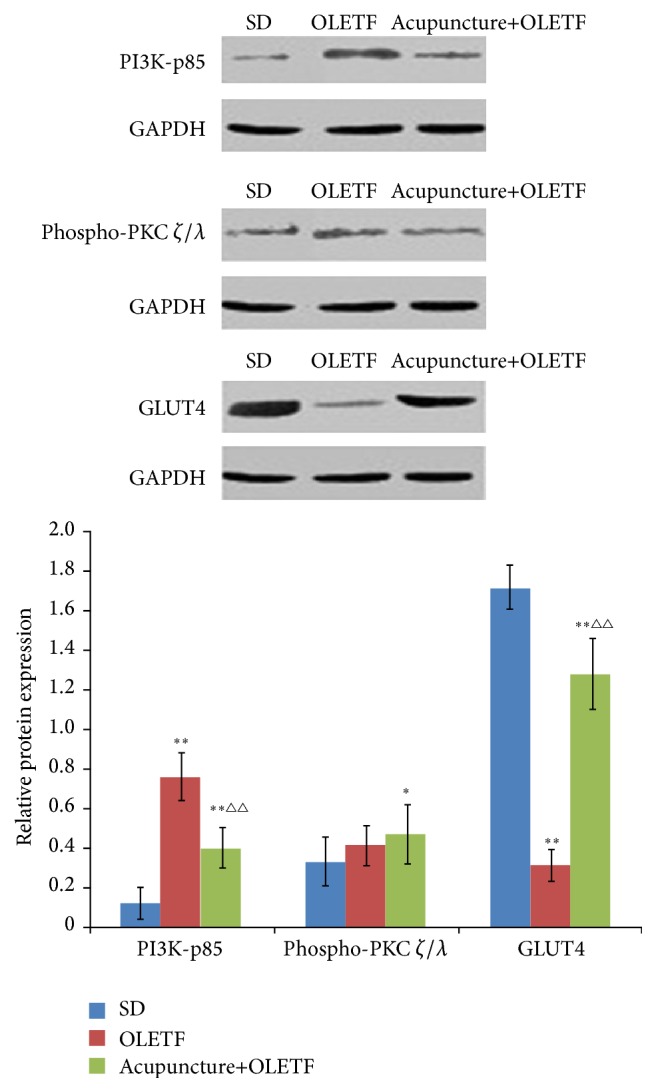
Effects of acupuncture on protein expressions of PI3K-p85, phospho-PKC *ζ*/*λ*, and GLUT4 in skeletal muscle of OLETF rats. Protein expression was determined by Western blot. GAPDH was used as an internal control. Data are shown as mean ± SD (*n* = 8 each group). ^*∗*^ *P* < 0.05 and ^*∗∗*^ *P* < 0.01* versus* SD group; ^△△^ *P* < 0.01* versus* OLETF group.

Protein expression of PI3K-p85 was significantly increased (*P* < 0.01) in OLETF rats compared to SD controls (Figure 3); importantly, this increase was attenuated by acupuncture treatment. In contrast, GLUT4 protein levels were reduced in the OLETF group compared to the control SD group (*P* < 0.01); again, we find that acupuncture attenuated this decrease in GLUT4 observed in the OLETF animals. Unlike PI3K-p85 and GLUT4, protein expression of phospho-PKC*ζ*/*λ* in the acupuncture+OLETF group was significantly increased compared to SD controls (*P* < 0.01), but not significantly altered compared to OLETF rats (*P* > 0.05).

## 4. Discussion

Acupuncture has been used as a remedy for a number of malignancies, including as a method of treatment to improve whole body metabolism. Several studies have found that acupuncture improves whole body glucose tolerance in human subjects of various ages and genders [4, 13]. It has been suggested that the improvements in whole body glucose tolerance are a result of increased body sensitivity that enhances glucose disposal from the body. Many of these same studies also showed that acupuncture could effectively reduce blood glucose concentration. However, it is plausible that acupuncture may also facilitate qualitative changes in skeletal muscle which contribute to the improvements in glucose metabolism. In the present investigation, we used genetically hyperglycemic rats as a model to evaluate the influence of acupuncture on skeletal muscle glucose uptake and transport. Furthermore, inclusion of the SD control group allowed us to assess specific adaptations in skeletal muscle glucose metabolism which occur in response to acupuncture. The results from this study show that acupuncture improves insulin resistance in OLETF rats, possibly via regulating expression of key insulin signaling related molecules.

Following acupuncture administration, we determined levels of plasma FPG, plasma FINS, HOMA-IR, and serum C-P as a measure of treatment efficacy. We also examined the influence of acupuncture treatment on expression levels of key insulin signaling components in skeletal muscle. OLETF rats exhibit significant insulin resistance with elevated levels of FPG, FINS, HOMA-IR, and C-P. Importantly, we find that many of these parameters are decreased to normal levels following acupuncture treatment. These findings raise the interesting possibility that improved insulin sensitivity following acupuncture administration may be due to alterations in components of the insulin signaling cascade. Along these lines, we find increased expression of IRS-1/2 after acupuncture, which likely contributes to improved rates of insulin-stimulated glucose uptake and transport. This is consistent with other reports showing that altered expression or activity of insulin-related molecules, for example, PI 3-kinase, is associated with altered rates of insulin-stimulated glucose uptake and transport [29, 30]. Also consistent with other reports, we find that 21 days of acupuncture administration increases PI 3-kinase activity in normal rodent skeletal muscle. Thus, acupuncture improves symptoms associated with insulin resistance possibly by altering the expression or activity of PI3K.

We next examined the effect of acupuncture on proteins downstream of PI3K in the insulin signal transduction cascade. Several earlier studies have shown that Akt phosphorylation is impaired in insulin-resistant tissue [31, 32]; this is in contrast to other reports stating that Akt phosphorylation is unaffected by insulin resistance [33, 34]. There is clearly some question surrounding Akt phosphorylation in response to insulin resistance, and there may very well be a tissue-type specific effect. To support this possibility, one study reported that chronic aerobic exercise increased insulin-stimulated Akt2 activity, but only within the red gastrocnemius in normal rodent skeletal muscle [35]. Importantly, it is Akt2 that accounts for the majority of insulin-regulated glucose uptake [36]. As such, these data clearly implicate Akt2 as the key isoform involved in this process and suggest that future efforts be directed toward understanding how activation of Akt2 is regulated. In this study, we observed decreased expression of Akt2 in OLETF rats compared to controls. We also show that acupuncture administration significantly attenuates the OLETF-mediated decrease in Akt2. Therefore, our findings suggest that insulin resistance in the OLETF model is associated with decreased Akt2 expression and that acupuncture may relieve some of the insulin-resistance related symptoms by restoring Akt2 expression back to normal levels.

PKC-*ζ*/*λ* is another downstream target of PI 3-kinase, which is suppressed in insulin-resistant skeletal muscle [31]; PKC-*ζ*/*λ* also appears to be negatively affected by high-fat feeding [37]. As such, we examined expression and activity of PKC-*ζ*/*λ* in skeletal muscle of OLETF rats following acupuncture treatment. Expression of PKC-*ζ*/*λ* at the mRNA level was minimally altered in the OLETF model or by acupuncture administration. Importantly, activation of PKC-*ζ*/*λ*, assessed by phosphospecific antibody staining by Western blot, was unaltered in the OLETF model with or without acupuncture. Thus, we conclude that signaling via PKC-*ζ*/*λ* is not a major contributing factor mediating glucose metabolism in skeletal muscle of OLETF rats.

While several studies have evaluated the effects of insulin-stimulated IRS-1/2-associated PI 3-kinase activity on glucose metabolism, it has recently been demonstrated that activation of PI 3-kinase alone may not be sufficient to fully account for insulin-stimulated glucose transport [38]. While insulin signaling has garnered some preliminary evaluation in skeletal muscle from genetically [39] and pharmacologically induced models [40] of insulin resistance, to the best of our knowledge, the present investigation is the first to assess insulin signaling activation in a normal rodent model of diabetes that were subjected to aerobic exercise. The specific role of insulin signaling in the regulation of insulin-stimulated glucose transport is unclear; however, the translocation of GLUT4 may be involved [41–43]. Consistent with this possibility, our findings show that acupuncture enhances expression of GLUT4 at both the protein and mRNA levels. This acupuncture-mediated increase may promote glucose transport and help alleviate some of the symptoms associated with insulin resistance.

The work we present here suggests that acupuncture improves insulin resistance in OLETF rats, possibly via regulating expression of key insulin signaling related molecules. However, we only managed to detect the expression of these proteins, among which some remained unchanged in their expression or activity during the experiment. As such, this represents an important limitation of our study. Additional work should be performed in which the effect of acupuncture on insulin resistance is determined in the presence of PI3K/Akt pathway inhibition, maybe, for example, with the use of small molecule inhibitors.

## 5. Conclusions

In summary, rodents subjected to 21 days of acupuncture display improved insulin sensitivity, which cooccurs with changes in expression of several components of the insulin signaling pathway, such as PI3K, Akt, and GLUT4. Thus, acupuncture may represent a useful therapeutic option for patients with T2DM.

## Figures and Tables

**Table 1:** Primers for real-time PCR.

Gene	Primer (5′-3′)	Product length (bp)
*β*-actin	F: CCTAGGCACCAGGGTGTGAT	122
	R: TTGGTGACAATGCCGTGTTC	

Akt2	F: CTCTGTAGCAGAATGCCAGC	103
	R: ATGGAAGGTCCTCTCGATGA	

aPKC*ζ*	F: CAGTGAAGGTGACCCTTGTA	75
	R: GGACGAAGTGCTCATCATCC	

aPKC*λ*	F: GCTCACTCCAGATGATGATG	120
	R: GGCAGTAAGCAGAATCAGAC	

GLUT4	F: GTTGGTCTCGGTGCTCTTAG	151
	R: GGCCACGATGGACACATAAC	

PI3K-p85*α*	F: ATACTTGATGTGGCTGACGC	110
	R: AATCCTCGTCATCGTCTACC	

IRS1	F: CTGGACGTCACAGGCAGAAT	110
	R: CGTGAGGTCCTGGTTGTGAA	

IRS2	F: GCCACCGTGGTGAAAGAGTA	183
	R: CCTGCCTCTTGGTTCCTTAT	

F: forward; R: reverse.

IRS1: insulin receptor substrate 1; IRS2: insulin receptor substrate 2; PI3K: phosphatidylinositol-3 kinase; PKC: protein kinase C; GLUT4: glucose transporter 4; Akt2: protein kinase B beta.

**Table 2:** Effects of acupuncture on plasma FPG, plasma FINS, serum C-P, and HOMA-IR in OLETF rats.

Group	FPG (mmol·L^−1^)	FINS (mU·L^−1^)	HOMA-IR	C-P (ng·L^−1^)
SD	9.26 ± 1.69	56.30 ± 0.74	1.36 ± 0.81	277.84 ± 10.96
OLETF	11.53 ± 2.43^*∗*^	59.53 ± 2.02^*∗∗*^	1.48 ± 0.86^*∗*^	305.41 ± 7.83^*∗∗*^
Acupuncture+OLETF	6.19 ± 0.93^*∗∗*△△^	56.67 ± 1.80^△△^	1.19 ± 0.66^*∗∗*△△^	286.44 ± 10.82^△△^

Data are shown as mean ± standard deviation (SD) (*n* = 8 each group).

^*∗*^ *P* < 0.05 and  ^*∗∗*^ *P* < 0.01* versus* SD group;  ^△△^ *P* < 0.01* versus* OLETF group.

FPG: fasting blood glucose; FINS: fasting insulin; C-P: C peptide; HOMA-IR: homeostasis model assessment-estimated insulin resistance; OLETF: Otsuka Long-Evans Tokushima Fatty; SD: Sprague-Dawley.
